# Predominance of atypical genotypes of *Toxoplasma gondii* in free-roaming chickens in St. Kitts, West Indies

**DOI:** 10.1186/s13071-017-2019-6

**Published:** 2017-02-27

**Authors:** Clare M. Hamilton, Patrick J. Kelly, Kenneth Boey, Tatiana M. Corey, Hieuhanh Huynh, Deidra Metzler, Isabelle Villena, Chunlei Su, Elisabeth A. Innes, Frank Katzer

**Affiliations:** 1 Moredun Research Institute, Pentlands Science Park, Bush Loan, Edinburgh, EH26 0PZ UK; 2 Ross University School of Veterinary Medicine, Island Main Road, West Farm, Saint Kitts and Nevis; 3 School of Applied Science, Temasek Polytechnic, 21 Tampines Avenue 1, Singapore, 529757 Singapore; 4 EA 3800, UFR Medecine, SFR CAP-SANTE, University of Reims Champagne Ardenne, Reims, France; 5 Laboratory of Parasitology, National Reference Centre on Toxoplasmosis, Hospital Maison Blanche, CHU Reims, Reims, France; 6 Department of Microbiology, University of Tennessee, 1414 W. Cumberland Avenue, Knoxville, TN 37996-0845 USA

**Keywords:** *Toxoplasma gondii*, Chickens, Genotyping, St. Kitts, West Indies

## Abstract

**Background:**

*Toxoplasma gondii* is a worldwide protozoan parasite of felids which can infect almost all warm-blooded animals, including humans. Free-roaming chickens are good indicators of environmental contamination with *T. gondii* oocysts because they feed from the ground. Previous research has demonstrated a high seroprevalence of *T. gondii* in domestic animals on St. Kitts but little is known about the genotypes circulating in the environment.

**Methods:**

Hearts and brains from 81 free-roaming chickens in St. Kitts were digested and inoculated into 243 Swiss Webster mice in a bioassay. DNA was extracted from digested chicken tissues and the brains of all mice, and screened for *T. gondii*. Positive samples were genotyped using restriction fragment length polymorphism. Chicken sera were also screened for *T. gondii* antibodies using a modified agglutination test (MAT).

**Results:**

Overall, 41% (33 out of 81) of chickens were positive for *T. gondii* either by serology and/or by PCR. Antibodies to *T. gondii* were detected by MAT in 32% (26 out of 81) of chickens, and *T. gondii* DNA was detected in mouse brains representing 26% (21 out of 81) of chickens. Genotyping of 21 DNA isolates, using polymorphisms at 10 loci, including SAG1, SAG2 (5′-3′ SAG2 and alt.SAG2), SAG3, BTUB, GRA6, c22-8, c29-2, L358, PK1 and Apico, revealed that 7 were ToxoDB genotype #141, 6 were #1 (Type II), 3 were #13, 3 were #265, one was #264 and one was #2 (Type III). Genotypes #13 and #141 appear to be more virulent.

**Conclusions:**

The results of this study highlight the greater genetic diversity of *T. gondii* circulating in the Caribbean region, with potentially different degrees of virulence to humans.

## Background

*Toxoplasma gondii* is a parasite of felids which can infect almost all warm-blooded animals, including humans [1]. Worldwide seroprevalences vary but it has been suggested that around one third of people have been infected with this parasite [2]. Transmission routes include ingestion of tissue cysts in infected meat, ingestion of sporulated oocysts (shed by felids) in contaminated food, water, or directly from the environment, and *via* vertical transmission, if a woman experiences a primary infection during pregnancy. Symptoms of toxoplasmosis are usually mild; however, immune-compromised people and congenitally infected infants can suffer severe, and even fatal, clinical signs [3].

Different factors may affect the severity of toxoplasmosis, including the infecting stage of the parasite, the size of inoculating dose, and the virulence of the infecting strain [4]. Historically, *T. gondii* was thought to have a clonal population structure comprising three dominant lineages (Types I, II and III) based on restriction fragment length polymorphism [5, 6]. However, there are indications of greater genetic variability with severe cases of toxoplasmosis in patients from South America being linked to genetically distinct strains of *T. gondii*, highlighting a greater genetic variability than previously thought [7, 8].

In 2002, Dubey et al. [9] initiated a worldwide survey of *T. gondii* in free-roaming chickens with the goal of characterising the genetic diversity of *T. gondii* on a global basis [10]. Free-roaming chickens are considered one of the most important hosts for studying the epidemiology of *T. gondii* because they feed from the ground and are therefore good indicators of environmental contamination with oocysts [10]. Also, the tissues of infected chickens are a good source of infection for cats, and potentially for humans if the meat is consumed undercooked. Strains of *T. gondii* have been characterized from free-roaming chickens in South America [9], Central America [11], the Caribbean [12], Asia [13], Africa [14] and Europe [15], and it is becoming apparent that isolates from South America, in particular Brazil, are genetically distinct [16].

Previous research in St. Kitts has demonstrated a high seroprevalence of *T. gondii* in domestic and feral cats [17, 18], as well as African green monkeys [19], livestock animals [20, 21] and dogs [22]. Genetic characterization of *T. gondii* isolated from livestock heart samples at the St. Kitts abattoir [21] and from feral cats [18] and dogs [22] revealed that the Type III genotype was predominant which was similar to other findings from Caribbean islands in this region [12], although atypical genotypes have also been reported [18, 21, 22].

In order to investigate the diversity of *T. gondii* genotypes circulating in the Caribbean, we isolated and genetically characterised viable isolates from free-roaming chickens in St. Kitts.

## Methods

### Animals

**Fig. 1:**
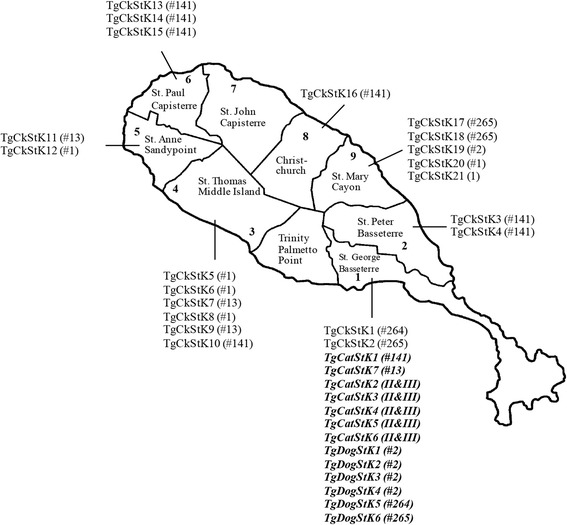
Map of St. Kitts showing nine areas where chickens were collected and the genotypes identified in each area from this study and previous studies (italicised bold) in St. Kitts [20, 24] (map adapted from mapsof.net)

This study was carried out between September 2014 and December 2014, following ethical approval from the Institutional Animal Care and Use Committee at RUSVM (Project Submission 30/020). Free-roaming chickens (*n* = 81) were collected, with permission from St. Kitts and Nevis Department of Agriculture and Chief Veterinary Officer, from 9 different locations (9 chickens from each) around the island (Fig. 1). Chickens were euthanized humanely by lethal injection and blood, heart and brain were collected from each. Sera were isolated as previously described [21] and stored at -20 °C until required. Hearts and brains were transferred to re-sealable bags prior to processing as described below.

### Serological examination

Aliquots of chicken sera were tested at the Toxoplasma Reference Laboratory (Reims, France) for reactive *T. gondii* antibodies using a modified agglutination test (MAT) [23]. Serum samples with an antibody titre of greater than or equal to 1:6 were considered positive.

### Bioassay of chicken tissues for *T. gondii*

Hearts and brains from all 81 chickens were digested in acid-pepsin solution as previously described [21], with the following modifications: the brain and heart from each chicken were pooled, homogenised in 25 ml (approximately five volumes (w/v)) 0.9% saline solution and then mixed with 25 ml (approximately five volumes (w/v)) acid-pepsin solution (which had been pre-warmed to 37 °C). After processing, the resulting pellet was resuspended in 10 ml PBS prior to being neutralized with 7.5 ml 1.2% sodium carbonate and centrifuged at 1200× *g* for 10 min. Two ml sterile saline solution containing 400 IU/ml penicillin and 400 μg/ml streptomycin were used to resuspend the final pellet.

Three Swiss CD-1® IGS mice (Charles River Laboratory, Boston, MA, USA) per chicken were each inoculated with 400 μl of homogenate, and 400 μl was kept for DNA extraction (see below). Mice were monitored twice daily and euthanized either when they showed clinical signs of *T. gondii* infection, or when they reached the end of the experiment (day 28 post inoculation). Immediately after euthanasia, mice were bled by cardiac puncture, and sera were isolated following centrifugation of clotted whole blood at 2000× *g* for 5 min and stored at -20 °C. Sera were tested for *T. gondii*-specific antibodies using an indirect ELISA (ID Screen® Toxoplasmosis Indirect Multi-species, IDvet, Montpellier, France) according to manufacturer’s instructions. Brain and right lung from each mouse were transferred to clean 2 ml tubes and stored at -80 °C for DNA extraction and genotyping.

### Detection of *T. gondii* DNA in digested chicken tissues

DNA was extracted from 400 μl of digested tissue homogenate per chicken using the Wizard® genomic DNA purification protocol (Promega Corporation, Southampton, UK) [24]. The final pellet of DNA was resuspended in 200 μl molecular-grade water and stored at -80 °C prior to use. Extraction controls were included within each group of DNA extractions as described previously [21]. PCR amplifications, targeting the ITS1 region between the 18S and 5.8S rRNA genes, were carried out in duplicate using conditions previously described [25]. In order to quantify the level of *T. gondii* DNA present in the digested chicken tissues (used as the mouse inocula), any samples positive by ITS1 PCR were also screened using a quantitative PCR, targeting the 529-bp repeat element, as previously described [21].

### Genetic characterisation of *T. gondii*

The whole brain from each mouse was homogenised (in 1 ml PBS) by passing tissue through an 18G needle followed by a 21G needle. DNA was extracted from 400 μl brain homogenate using the method described above. All 243 samples were initially examined for the DNA of *T. gondii* using the nested ITS1 PCR described above. Samples which were positive were then genotyped utilising a multiplex nested PCR-RFLP targeting 10 genetic markers, including SAG1, SAG2 (5′-3′ SAG2 and alt.SAG2), SAG3, BTUB, GRA6, c22-8, c29-2, L358, PK1 and Apico. PCR-RFLP conditions for all markers were carried out as previously described [21]. Typing profiles were determined using RFLP banding profiles of reference strains [26].

In order to compare genotypes of *T. gondii* isolated from mouse tissues and chicken tissues, PCR-RFLP was carried out as described above using DNA extracted from the digested chicken tissues (mouse inoculum).

### Statistical analysis

A two-way ANOVA was used to investigate the effects of genotype and quantity of *T. gondii* DNA in the inocula on the percentage of mouse mortality. A separate one-way ANOVA with LSD post-hoc tests was used to further investigate the effect of genotype on the percentage of mouse mortality. A *P* value of < 0.05 was deemed significant. The statistical software package Minitab 17 was used for all statistical analyses.

## Results

Overall, 33 out of 81 (41%) chickens were positive for *T. gondii* either by serology (MAT-positive) and/or by ITS1 PCR, whereby *T. gondii* DNA was detected either in digested chicken tissues or in the brain tissues of inoculated mice (Table 1). Antibodies to *T. gondii* were detected by MAT in 32% (26 of 81) of chickens, with titres of 1:6 in 10, 1:10 in 5, 1:25 in 3, 1:50 in 4, 1:100 in 1, 1:400 in 1, 1:800 in 1, and 1:3200 in 1 chicken (Table 1). *Toxoplasma gondii* DNA was detected in the homogenised brain and heart tissues (used as the mouse inoculum) of 28% (23 of 81) of chickens (Table 1). Of these, 5 had an MAT titre of 1:6, 3 had a titre of 1:10, 2 had a titre of 1:25, 3 had a titre of 1:50, 1 had a titre of 1:400, 1 had a titre of 1:800, 1 had a titre of 1:3200, and 7 were seronegative with a titre of less than or equal to 1:3. *Toxoplasma gondii* DNA was detected in mouse brains representing 26% (21 of 81) chickens, including 4 chickens with an MAT titre of 1:6, 2 with a titre of 1:10, 2 with a titre of 1:25, 4 with a titre of 1:50, 1 with a titre of 1:400, 1 with a titre of 1:800, 1 with a titre of 1:3200, and also 6 seronegative chickens with titres of less than or equal to 1:3.

**Table 1:** Summary of *Toxoplasma gondii* isolation from free-roaming chickens in St. Kitts

Location	Parish	MAT POS^a^	ITS1 POS^b^	Bioassay POS^c^	MAT and/or PCR POS^d^
1	St. Georges Basseterre	2/9	2/9	2/9	3/9
2	St. Peters Basseterre	2/9	3/9	2/9	3/9
3	St. Thomas Middle Island	3/9	5/9	5/9	6/9
4	St. Thomas Middle Island	6/9	0/9	1/9	6/9
5	St. Anne Sandy Point	2/9	2/9	2/9	2/9
6	St. Paul Capisterre	4/9	3/9	3/9	4/9
7	St. John Capisterre	1/9	0/9	0/9	1/9
8	Christchurch	2/9	2/9	1/9	2/9
9	St. Mary Cayon	4/9	6/9	5/9	6/9
Total		26/81 (32%)	23/81 (28%)	21/81 (26%)	33/81 (41%)

^a^Number of chickens seropositive/number of chickens tested

^b^Number of chickens positive for *T. gondii* DNA (chicken tissues positive by ITS1 PCR)/number of chickens tested

^c^Number of chickens positive by bioassay (where brain tissue from at least one mouse out of 3 was positive by ITS1 PCR)/number of chickens tested

^d^Number of chickens *T. gondii*-positive by either serology (MAT) and/or PCR (either chicken tissues or mouse tissues)/Number of chickens tested

Thirty out of the 243 mice inoculated [representing 12 out of 81 chickens (15%)] had to be euthanized before the end of the experiment due to clinical signs of toxoplasmosis (Table 2).

**Table 2:** Burden of *Toxoplasma gondii* DNA in chicken tissues (used as mouse inocula)

Chicken ID^a^	qPCR	DNA (pg)	No. of mice infected (out of 3 inoculated)	No. of mice euthanized early due to infection (% mortality)^b^
1.6	Pos	0.3	3	0 (0)
1.8	Pos	1.5	3	0 (0)
2.1	Pos	11.1	3	3 (100)
2.3	Pos	1.8	3	3 (100)
2.7	Pos	0.04	0	0 (0)
3.2	Neg	0.0	3	0 (0)
3.4	Pos	0.3	3	0 (0)
3.5	Pos	0.7	3	3 (100)
3.6	Pos	0.1	3	1 (33.3)
3.8	Pos	5.6	3	2 (66.7)
5.1	Pos	6.3	3	3 (100)
5.2	Pos	13.6	3	2 (66.7)
6.1	Pos	21.6	3	3 (100)
6.2	Pos	1.1	3	3 (100)
6.6	Pos	1.9	3	3 (100)
8.1	Neg	0.0	0	0 (0)
8.5	Pos	0.07	3	3 (100)
9.1	Pos	0.2	2	0 (0)
9.2	Pos	2.5	3	0 (0)
9.3	Pos	1.7	3	0 (0)
9.5	Neg	0.0	3	0 (0)
9.8	Pos	0.3	3	0 (0)
9.9	Neg	0.0	0	0 (0)

*Abbreviations*: Neg, *T. gondii* qPCR-negative; Pos, *T. gondii* qPCR-positive

^a^Chickens which were positive by ITS1 PCR

^b^One mouse missing from table, it was inoculated with chicken tissues which were negative by ITS1 PCR and qPCR

Genotyping of 21 isolates from chickens indicated that 14 (67%) were atypical, 6 were ToxoDB genotype #1 (Type II) and one was genotype #2 (Type III) (Table 3 and Fig. 1). Of the 14 atypical genotypes, 7 were ToxoDB genotype #141, 3 were genotype #13, 3 were genotype #265 and 1 was genotype #264. No Type I genotypes were identified and no mixed infections were found. The genetic data were based on DNA from one mouse in each group of 3 mice inoculated with chicken tissues. For the two chickens which were positive by PCR but negative in the bioassay, no products could be amplified at any loci.

**Table 3:** Genetic characterisation of *T. gondii* isolated from chickens in St. Kitts

Chicken ID^a^	MAT titre^b^	Source of DNA^c^	No. of mice infected^d^	Genetic characterisation												
				Isolate ID	SAG1	SAG2 (5’ and 3’)	Alt. SAG2	SAG3	BTUB	GRA6	c22-8	c29-2	L358	PK1	Apico	ToxoDB Genotype
1.6	1:6	Mouse	3 (0)	TgCkStK1	II or III	II	II	II	III	I	II	I	I	II	I	#264
		Chicken			II or III	II	II	II	III	na	II	na	I	II	I	
1.8	1:3	Mouse	3 (0)	TgCkStK2	II or III	II	II	III	II	II	II	III	III	II	III	#265
		Chicken			na	na	II	III	na	II	na	na	III	II	III	
2.1	1:800	Mouse	3 (3)	TgCkStK3	II or III	III	III	III	III	III	III	III	III	I	III	#141
		Chicken			II or III	III	III	III	III	III	III	III	III	I	III	
2.3	1:50	Mouse	3 (3)	TgCkStK4	II or III	III	III	III	III	III	III	III	III	I	III	#141
		Chicken			II or III	III	III	III	III	III	III	III	III	I	III	
3.2	0	Mouse	3 (0)	TgCkStK5	II or III	II	II	II	II	II	II	II	II	II	II	#1
		Chicken			na	II	II	II	II	na	na	II	II	II	na	
3.4	0	Mouse	3 (0)	TgCkStK6	II or III	II	II	II	II	II	II	II	II	II	II	#1
		Chicken			II or III	II	II	II	II	II	II	II	II	II	II	
3.5	1:6	Mouse	3 (3)	TgCkStK7	I	I	I	I	I	III	II	III	III	I	III	#13
		Chicken			I	I	I	I	I	III	II	III	III	I	na	
3.6	0	Mouse	3 (1)	TgCkStK8	II or III	II	II	II	II	II	II	II	II	II	II	#1
		Chicken			II or III	II	na	II	II	na	II	II	II	II	II	
3.8	1:10	Mouse	3 (2)	TgCkStK9	I	I	I	I	I	III	II	III	III	I	III	#13
		Chicken			I	I	I	I	I	III	II	III	III	I	III	
4.4	1:50	Mouse	3 (1)	TgCkStK10	II or III	III	III	III	III	III	III	III	III	I	III	#141
		Chicken			na	na	na	na	na	na	na	na	na	na	na	
5.1	1:3200	Mouse	3 (3)	TgCkStK11	I	I	I	I	I	III	II	III	III	I	III	#13
		Chicken			I	I	I	I	I	III	II	III	III	I	III	
5.2	1:25	Mouse	3 (2)	TgCkStK12	II or III	II	II	II	II	II	II	II	II	II	II	#1
		Chicken			II or III	II	II	II	II	II	II	II	II	II	II	
6.1	1:50	Mouse	3 (3)	TgCkStK13	II or III	III	III	III	III	III	III	III	III	I	III	#141
		Chicken			II or III	III	III	III	III	III	III	III	III	I	III	
6.2	1:6	Mouse	3 (3)	TgCkStK14	II or III	III	III	III	III	III	III	III	III	I	III	#141
		Chicken			II or III	III	III	III	III	III	III	III	III	I	III	
6.6	1:50	Mouse	3 (3)	TgCkStK15	II or III	III	III	III	III	III	III	III	III	I	III	#141
		Chicken			II or III	III	na	III	III	III	III	III	III	I	III	
8.5	1:25	Mouse	3 (3)	TgCkStK16	II or III	III	III	III	III	III	III	III	III	I	III	#141
		Chicken			II or III	III	III	III	III	III	III	na	III	I	na	
9.1	1:3	Mouse	2 (0)	TgCkStK17	II or III	II	II	III	II	II	II	III	III	II	III	#265
		Chicken			II or III	II	II	III	II	II	II	III	III	II	III	
9.2	1:10	Mouse	3 (0)	TgCkStK18	II or III	II	II	III	II	II	II	III	III	II	III	#265
		Chicken			II or III	II	II	III	II	II	II	III	III	II	III	
9.3	1:6	Mouse	3 (0)	TgCkStK19	II or III	II	III	III	III	III	III	III	III	III	III	#2
		Chicken			II or III	II	III	III	III	III	III	na	III	III	na	
9.5	1:3	Mouse	3 (0)	TgCkStK20	II or III	II	II	II	II	II	II	II	II	II	II	#1
		Chicken			na	na	II	na	na	na	II	II	II	na	II	
9.8	1:4000	Mouse	3 (0)	TgCkStK21	II or III	II	II	II	II	II	II	II	II	II	II	#1
		Chicken			II or III	II	II	II	II	II	II	II	II	II	II	

*Abbreviations*: *MAT* modified agglutination test, *na* not amplified

^a^Location number followed by chicken number

^b^ *T. gondii* MAT titre for chicken sera (1:6 or above was considered positive)

^c^DNA extracted from mouse brains or digested chicken tissues

^d^Out of 3 mice inoculated (number of mice euthanized early due to clinical toxoplasmosis)

Twenty one (21) chickens which tested *T. gondii*-positive by ITS1 PCR and also by bioassay (i.e. at least one out of three mice were positive for *T. gondii*) had their tissues tested by qPCR to quantify the amount of *T. gondii* DNA present. When comparing the percentage mortality of mice in the bioassay to the quantity of *T. gondii* DNA and the genotype present in the inocula, there was no significant effect of *T. gondii* DNA quantity (*F*_(3,12)_ = 1.5, *P* = 0.265) but there was a significant effect of genotype (*F*_(5,12)_ = 11.91, *P* < 0.0001). *Post-hoc* Fisher pairwise comparisons revealed that mice inoculated with genotypes #141 and #13 had significantly higher levels of mortality (*P* ≤ 0.05).

## Discussion

The results of this study demonstrate a high prevalence of *T. gondii* in free-roaming chickens in St. Kitts and also reveal a greater genetic diversity of strains circulating on the island, with potentially different degrees of virulence to humans. Genotyping of 21 isolates in the present study revealed a predominance of atypical genotypes, the majority of which have previously been reported in St. Kitts. Genotypes #264 and #265 were only very recently isolated from feral dogs in St. Kitts and were avirulent for mice [22]. Genotypes #141 and #13 were previously isolated from feral cats in St. Kitts (TgCatStK1 and TgCatStK7, respectively) [18] and, therefore, further attests to the widespread environmental contamination with *T. gondii* oocysts. Genotype #141 has also been isolated from a fox in Pennsylvania, USA, and, notably, has been demonstrated to be acutely virulent to outbred mice even at low infection levels (10 tachyzoites) [27]. Genotype #13 has previously been isolated from chickens [16] and a goat [28] in Brazil, chickens [29] and a dog [30] in Grenada and a howler monkey in Brazil [31]. While these studies report that genotype #13 is avirulent for mice, in the present study 8 out of 9 mice inoculated with genotype #13 had to be euthanized due to clinical signs of toxoplasmosis. This may have been attributable to the quantity of *T. gondii* DNA present in the inocula rather than a reflection of virulence of the parasite but statistical analyses demonstrated that quantity of *T. gondii* DNA in the inocula was not a significant factor in mortality whereas genotype was, with significantly more euthanasias being recorded for mice inoculated with genotypes #13 and #141.

Strains which are virulent for mice are not necessarily virulent for humans but previous studies have reported isolating the Caribbean 1 isolate from an AIDS patient from Martinique where toxoplasmic encephalitis was recorded at autopsy [32], an AIDS patient from Guadeloupe who presented with toxoplasmic lymphadenopathy and had a previous history of toxoplasmic encephalitis [32], and a bone marrow transplant patient from Guadeloupe who died from pulmonary toxoplasmosis [32]. The Caribbean 1 isolate has been defined using microsatellite markers [33, 34] but in the case of toxoplasmic lymphadenopathy described above, the isolate has been genotyped using PCR-RFLP and defined as ToxoDB genotype #13 (Chunlei Su, personal communication). It is also possible that the other Caribbean 1 isolates (FDF-2007-HEN and CCH-2005-REN) are genotype #13 (Daniel Ajzenberg, personal communication). Further studies are currently underway in our laboratory to investigate the pathogenicity of the isolates from St. Kitts; however, the presence of potentially virulent strains circulating on the island is a public health issue and officials should take note.

Identifying 7 different genotypes from only 21 isolates would indicate that the overall genetic diversity of *T. gondii* on St. Kitts is relatively high. It appears that the Caribbean region is more in line with the diversity found in Central and South America than in North America and Europe. A recent study looking at geographical patterns of *T. gondii* genetic diversity revealed 156 different genotypes from 646 South/Central American isolates (24%) but only 9 genotypes from 64 European isolates (14%), 10 from 102 Asian isolates (10%), 13 from 141 African isolates (9%) and 40 from 501 North American isolates (8%) [35].

Worldwide serological prevalence of *T. gondii* in chickens varies widely (2–100%) and is dependent on the method of detection used and the source of chickens [10]. At present, there is no “gold standard” serological test for chickens although the MAT is considered the most specific [36–38]. In the present study, MAT antibody titres of ≥ 1:6 were detected in 32% of chickens tested indicating that these animals have become infected with *T. gondii* in their environment in St. Kitts. The seroprevalence rate in St. Kitts is lower than that reported in the fellow Caribbean island of Grenada (52%) [12]; however, neither study documented the age of the animals which could influence results, since older animals are more likely to be seropositive for *T. gondii* [39, 40]. The domestic [17] and feral [18] cat populations in St. Kitts are known to have high *T. gondii* seroprevalence rates, suggesting that contamination with oocysts could be widespread on the island. Recent studies of dogs and livestock in St. Kitts and Nevis reported seroprevalences of 42% (dogs) [22], 26% (sheep) and 34% (goats) [21]. Together with the results of the current study, where chickens were collected from all over the island, it is evident that there is widespread contamination with *T. gondii* oocysts in St. Kitts. This could pose a potential public health issue given that sporulated, infective, *T. gondii* oocysts can survive in the environment for over a year [41, 42].

The results of the MAT (32% seropositive) were not too dissimilar to the results of the bioassay (26% *Toxoplasma*-positive) and ITS1 PCR on the digested tissues (28% *Toxoplasma*-positive) suggesting that the MAT is a good serological indicator of *T. gondii* infection. However, of seven chickens which were deemed seronegative by MAT (titre ≤ 1:3), six were positive by bioassay (mice were positive by ITS1 PCR and/or serology) and all seven were ITS1 PCR positive (DNA extracted from homogenised chicken tissues). Previous reports have demonstrated that *T. gondii* could be isolated from defined seronegative chickens [12, 39] indicating that MAT is perhaps not as sensitive at detecting early stages of infection when antibody levels are lower, or it could indicate that the chickens were persistently infected and the antibody levels had declined [43]. A previous study investigating the persistence of *T. gondii* in tissues of experimentally infected poultry reported decreasing antibody titres in chickens four weeks post-infection [43]. This would suggest that seroprevalence studies may underestimate the true prevalence of *T. gondii* in free-roaming chickens.

It is of note that nine chickens tested positive by MAT but were negative in the bioassay and ITS1 PCR. This discrepancy may have been due to the inhomogeneous distribution of *T. gondii* tissue cysts in the digested tissues or because the tissues were no longer harbouring cysts. Chickens are clinically resistant to *T. gondii* [10] and in a recent study of experimentally infected chickens, only 4 out of 192 tested positive by PCR and only one tissue (heart) was still found to be positive by ten weeks post-infection [43].

Although we did not test breast muscle or thigh muscle, it is possible that these tissues may have contained viable *T. gondii* tissue cysts [10, 44], highlighting a possible public health risk to the inhabitants of St. Kitts who handle or consume locally produced chicken meat [10]. Also, given the evidence for environmental contamination with oocysts, there is also a potential for transmission to the population through contaminated water [45]. Until very recently, there was no data on *T. gondii* infection rates in people in St. Kitts. Dubey et al. [22] conducted a study on pregnant women in the Caribbean and reported that 8 out of 44 (18%) women in St. Kitts were seropositive for *T. gondii* by MAT. This was comparable to Bermuda (18%) but lower than other Caribbean islands, including Dominica (59%), St. Vincent and the Grenadines (54%), St. Lucia (53%), Grenada (37%), Montserrat (33%), and Antigua and Barbuda (32%) [22]. Chicken is a popular meat in the Caribbean and is frequently eaten barbequed which could pose an infection risk if the meat is infected and not cooked thoroughly. However, the tendency in this region is to consume meat (not just chicken) well-done so the risk of infection is likely to be low, since tissue cysts are killed at temperatures of over 67 °C for 10 min [46]. People may be at more risk of infection from a lack of hand washing after handling raw infected meat, particularly in the case of backyard farming where chickens are killed at home and their viscera may be improperly handled and disposed of [10].

## Conclusion

In summary, there is a greater genetic diversity of *T. gondii* circulating in the Caribbean region, with potentially different degrees of virulence to humans. The study also confirms that the environment in St. Kitts is contaminated with *T. gondii* oocysts and highlights chicken meat as a potentially important source of *T. gondii* infection if it is handled with poor hygiene standards or if it is consumed undercooked.

## References

[1] Dubey JP (2008). The history of *Toxoplasma gondii* - the first 100 years. J Eukaryot Microbiol.

[2] Pappas G, Roussos N, Falagas ME (2009). Toxoplasmosis snapshots: global status of *Toxoplasma gondii* seroprevalence and implications for pregnancy and congenital toxoplasmosis. Int J Parasitol.

[3] Tenter AM, Heckeroth AR, Weiss LM (2000). *Toxoplasma gondii*: from animals to humans. Int J Parasitol.

[4] Scientific opinion of the Panel on Biological Hazards on a request from EFSA on surveillance and monitoring of Toxoplasma in humans, food and animals. The EFSA Journal 2007;583:1-64.

[5] Howe DK, Sibley LD (1995). *Toxoplasma gondii* comprises three clonal lineages: correlation of parasite genotype with human disease. J Infect Dis.

[6] Ajzenberg D, Banuls AL, Tibayrenc M, Darde ML (2002). Microsatellite analysis of *Toxoplasma gondii* shows considerable polymorphism structured into two main clonal groups. Int J Parasitol.

[7] Ferreira IM, Vidal JE, de Mattos CC, de Mattos LC, Qu D, Su C, Pereira-Chioccola VL (2011). *Toxoplasma gondii* isolates: multilocus RFLP-PCR genotyping from human patients in Sao Paulo State, Brazil identified distinct genotypes. Exp Parasitol.

[8] Carme B, Demar M, Ajzenberg D, Darde ML (2009). Severe acquired toxoplasmosis caused by wild cycle of *Toxoplasma gondii*, French Guiana. Emerg Infect Dis.

[9] Dubey JP, Graham DH, Blackston CR, Lehmann T, Gennari SM, Ragozo AM (2002). Biological and genetic characterisation of *Toxoplasma gondii* isolates from chickens (*Gallus domesticus*) from Sao Paulo, Brazil: unexpected findings. Int J Parasitol.

[10] Dubey JP (2010). *Toxoplasma gondii* infections in chickens (*Gallus domesticus*): prevalence, clinical disease, diagnosis and public health significance. Zoonoses Public Health.

[11] Dubey JP, Lopez B, Alvarez M, Mendoza C, Lehmann T (2005). Isolation, tissue distribution, and molecular characterization of *Toxoplasma gondii* from free-range chickens from Guatemala. J Parasitol.

[12] Dubey JR, Bhaiyat MI, de Allie C, Macpherson CN, Sharma RN, Sreekumar C (2005). Isolation, tissue distribution, and molecular characterization of *Toxoplasma gondii* from chickens in Grenada, West Indies. J Parasitol.

[13] Sreekumar C, Graham DH, Dahl E, Lehmann T, Raman M, Bhalerao DP (2003). Genotyping of *Toxoplasma gondii* isolates from chickens from India. Vet Parasitol.

[14] Dubey JP, Karhemere S, Dahl E, Sreekumar C, Diabate A, Dabire KR (2005). First biologic and genetic characterization of *Toxoplasma gondii* isolates from chickens from Africa (Democratic Republic of Congo, Mali, Burkina Faso, and Kenya). J Parasitol.

[15] Dubey JP, Vianna MC, Sousa S, Canada N, Meireles S, Correia da Costa JM (2006). Characterization of *Toxoplasma gondii* isolates in free-range chickens from Portugal. J Parasitol.

[16] Dubey JP, Velmurugan GV, Chockalingam A, Pena HF, de Oliveira LN, Leifer CA (2008). Genetic diversity of *Toxoplasma gondii* isolates from chickens from Brazil. Vet Parasitol.

[17] Moura L, Kelly P, Krecek RC, Dubey JP (2007). Seroprevalence of *Toxoplasma gondii* in cats from St. Kitts, West Indies. J Parasitol.

[18] Dubey JP, Moura L, Majumdar D, Sundar N, Velmurugan GV, Kwok OC, Kelly P (2009). Isolation and characterization of viable *Toxoplasma gondii* isolates revealed possible high frequency of mixed infection in feral cats (*Felis domesticus*) from St. Kitts, West Indies. Parasitology.

[19] Hamilton CM, Katzer F, Beierschmitt A, Soto E, Innes EA, Kelly PJ (2014). First report of *Toxoplasma gondii* seroprevalence in wild-caught Caribbean African green monkeys. Parasit Vectors.

[20] Hamilton CM, Katzer F, Innes EA, Kelly PJ (2014). Seroprevalence of *Toxoplasma gondii* in small ruminants from four Caribbean islands. Parasit Vectors.

[21] Hamilton CM, Kelly PJ, Bartley PM, Burrells A, Porco A, Metzler D (2015). *Toxoplasma gondii* in livestock in St. Kitts and Nevis Parasit Vectors.

[22] Dubey JP, Verma SK, Villena I, Aubert D, Geers R, Su C (2016). Toxoplasmosis in the Caribbean islands: literature review, seroprevalence in pregnant women in ten countries, isolation of viable *Toxoplasma gondii* from dogs from St. Kitts, West Indies with report of new *T. gondii* genetic types. Parasitol Res.

[23] Dubey JP, Desmonts G (1987). Serological responses of equids fed *Toxoplasma gondii* oocysts. Equine Vet J.

[24] Katzer F, Canton G, Burrells A, Palarea-Albaladejo J, Horton B, Bartley PM (2014). Immunization of lambs with the S48 strain of *Toxoplasma gondii* reduces tissue cyst burden following oral challenge with a complete strain of the parasite. Vet Parasitol.

[25] Burrells A, Bartley PM, Zimmer IA, Roy S, Kitchener AC, Meredith A (2013). Evidence of the three main clonal *Toxoplasma gondii* lineages from wild mammalian carnivores in the UK. Parasitology.

[26] Burrells A, Opsteegh M, Pollock KG, Alexander CL, Chatterton J, Evans R (2016). The prevalence and genotypic analysis of *Toxoplasma gondii* from individuals in Scotland, 2006-2012. Parasit Vectors.

[27] Dubey JP, Van Why K, Verma SK, Choudhary S, Kwok OC, Khan A (2014). Genotyping *Toxoplasma gondii* from wildlife in Pennsylvania and identification of natural recombinants virulent to mice. Vet Parasitol.

[28] Ragozo AM, Yai LE, Oliveira LN, Dias RA, Goncalves HC, Azevedo SS (2009). Isolation of *Toxoplasma gondii* from goats from Brazil. J Parasitol.

[29] Rajendran C, Su C, Dubey JP (2012). Molecular genotyping of *Toxoplasma gondii* from Central and South America revealed high diversity within and between populations. Infect Genet Evol.

[30] Dubey JP, Tiwari K, Chikweto A, Deallie C, Sharma R, Thomas D (2013). Isolation and RFLP genotyping of *Toxoplasma gondii* from the domestic dogs (*Canis familiaris*) from Grenada, West Indies revealed high genetic variability. Vet Parasitol.

[31] Pena HF, Marvulo MF, Horta MC, Silva MA, Silva JC, Siqueira DB (2011). Isolation and genetic characterisation of *Toxoplasma gondii* from a red-handed howler monkey (*Alouatta belzebul*), a jaguarundi (*Puma yagouaroundi*), and a black-eared opossum (*Didelphis aurita*) from Brazil. Vet Parasitol.

[32] Ajzenberg D, Yera H, Marty P, Paris L, Dalle F, Menotti J (2009). Genotype of 88 *Toxoplasma gondii* isolates associated with toxoplasmosis in immunocompromised patients and correlation with clinical findings. J Infect Dis.

[33] Ajzenberg D, Lamaury I, Demar M, Vautrin C, Cabie A, Simon S (2016). Performance testing of PCR assay in blood samples for the diagnosis of toxoplasmic encephalitis in AIDS patients from the French Departments of America and genetic diversity of *Toxoplasma gondii*: A prospective and multicentric study. PLoS Negl Trop Dis.

[34] Ajzenberg D, Banuls AL, Su C, Dumetre A, Demar M, Carme B, Darde ML (2004). Genetic diversity, clonality and sexuality in *Toxoplasma gondii*. Int J Parasitol.

[35] Shwab EK, Zhu XQ, Majumdar D, Pena HF, Gennari SM, Dubey JP, Su C (2014). Geographical patterns of *Toxoplasma gondii* genetic diversity revealed by multilocus PCR-RFLP genotyping. Parasitology.

[36] Dubey JP, Laurin E, Kwowk OC (2016). Validation of the modified agglutination test for the detection of *Toxoplasma gondii* in free-range chickens by using cat and mouse bioassay. Parasitology.

[37] Dubey JP, Graham DH, Dahl E, Sreekumar C, Lehmann T, Davis MF, Morishita TY (2003). *Toxoplasma gondii* isolates from free-ranging chickens from the United States. J Parasitol.

[38] Casartelli-Alves L, Boechat VC, Macedo-Couto R, Ferreira LC, Nicolau JL, Neves LB (2014). Sensitivity and specificity of serological tests, histopathology and immunohistochemistry for detection of *Toxoplasma gondii* infection in domestic chickens. Vet Parasitol.

[39] Zhao G, Shen B, Xie Q, Xu LX, Yan RF, Song XK, Hassan IA, Li XR (2012). Detection of *Toxoplasma gondii* in free-range chickens in China based on circulating antigens and antibodies. Vet Parasitol.

[40] Katzer F, Brulisauer F, Collantes-Fernandez E, Bartley PM, Burrells A, Gunn G (2011). Increased *Toxoplasma gondii* positivity relative to age in 125 Scottish sheep flocks; evidence of frequent acquired infection. Vet Res.

[41] Frenkel JK, Ruiz A, Chinchilla M (1975). Soil survival of toxoplasma oocysts in Kansas and Costa Rica. Am J Trop Med Hyg.

[42] Dubey JP (1998). *Toxoplasma gondii* oocyst survival under defined temperatures. J Parasitol.

[43] Geuthner AC, Koethe M, Ludewig M, Pott S, Schares G, Daugschies A, Bangoura B (2014). Persistence of *Toxoplasma gondii* tissue stages in poultry over a conventional fattening cycle. Parasitology.

[44] Hill DE, Dubey JP: Toxoplasmosis. US Geological Survey Circular 1389 2014. http://dx.doi.org/10.3133/cir1389.

[45] Wells B, Shaw H, Innocent G, Guido S, Hotchkiss E, Parigi M (2015). Molecular detection of *Toxoplasma gondii* in water samples from Scotland and a comparison between the 529bp real-time PCR and ITS1 nested PCR. Water Res.

[46] Dubey JP, Kotula AW, Sharar A, Andrews CD, Lindsay DS (1990). Effect of high temperature on infectivity of *Toxoplasma gondii* tissue cysts in pork. J Parasitol.

